# Acute-phase proteins as biomarkers of inflammation in HIV patients with latent tuberculosis: a prospective study

**DOI:** 10.3389/fimmu.2025.1551775

**Published:** 2025-04-30

**Authors:** Nathella Pavan Kumar, Rajamariyam M, Arul Nancy, Bubby S. Kumar, Janakiram M, Shaik Fayaz Ahamed, Vinod Kumar, Vijila S, Sekar S, Kuralmozhi R, Dhanaraj Baskaran, Syed Hissar, Subash Babu

**Affiliations:** ^1^ Department of Immunology, Indian Council of Medical Research-National Institute for Research in Tuberculosis, Chennai, India; ^2^ ICER, National Institutes of Health-National Institute for Research in Tuberculosis-International Center for Excellence in Research, Chennai, India; ^3^ Monitoring & Evaluation, Care Support & Treatment, Tamil Nadu State AIDS Control Society, Chennai, India; ^4^ Respiratory Medicine, Govt. Hospital of Thoracic Medicine, Chennai, India; ^5^ ART Center Rajiv Gandhi Govt. General Hospital, Chennai, India; ^6^ Department of Clinical Research, Indian Council of Medical Research-National Institute for Research in Tuberculosis, Chennai, India

**Keywords:** acute-phase proteins, biomarkers, HIV, LTBI, isoniazid preventive treatment (IPT)

## Abstract

**Introduction:**

Tuberculosis (TB) remains the primary cause of death among individuals infected with HIV, increasing the risk of contracting TB by up to 26 times. This co-infection complicates the diagnosis and treatment of TB, ultimately affecting outcomes adversely. Acute-phase proteins (APPs), markers of inflammation, are significantly elevated during infections and serve as critical indicators of inflammation resulting from infectious diseases.

**Methods:**

In this prospective study, HIV-positive individuals at antiretroviral therapy (ART) clinics were screened for latent tuberculosis infection (LTBI) before starting isoniazid (INH) prophylaxis. Initially, 101 patients were enrolled, with 71 completing a six-month follow-up on INH prophylaxis. LTBI was diagnosed using QuantiFERON-TB Gold plus, categorizing participants as HIV-positive with LTBI (n=30) and HIV-positive without LTBI (n=71).

**Results:**

Plasma levels of alpha-2-macroglobulin (A2M), C-reactive protein (CRP), serum amyloid P (SAP), haptoglobin, ferritin, soluble transferrin receptor (sTFR), apotransferrin, hepcidin, and S100A8/A9 were assessed using multiplex and quantikine assays.At baseline, levels of A2M, CRP, SAP, ferritin, hepcidin, and S100A9 were significantly elevated in HIV-positive patients with LTBI compared to those without LTBI (A2M, p=0.005; CRP, p<0.001; SAP, p=0.0006; ferritin, p<0.001; hepcidin, p=0.001; S100A9, p=0.001). Following six months of INH prophylaxis, significant reductions in these markers were observed in both groups, suggesting a reduction in inflammation.

**Discussion:**

Our findings indicate that a baseline profile of APPs can effectively reflect the inflammatory status of HIV patients with LTBI. These inflammatory markers tend to decrease following effective INH treatment, underscoring their potential utility in monitoring disease progression and treatment response in this vulnerable population.

## Introduction

Human Immunodeficiency Virus (HIV) remains a significant global health issue, having claimed over 40.4 million lives according to the World Health Organization. HIV specifically targets and depletes CD4+ T cells, leading to acquired immunodeficiency syndrome (AIDS) after an initial asymptomatic period during which the virus replicates at low levels (1, 2). This depletion severely impairs immune functions, increasing vulnerability to opportunistic infections such as tuberculosis (TB), the leading cause of death among those infected with HIV (1, 3). Research has shown that HIV-infected individuals have a 26-fold increased risk of reactivating latent TB infection (LTBI), with reactivation rates as high as 5-15% annually among HIV-positive individuals (4–6). This syndemic relationship underscores the critical need to understand the immunological interplay between HIV and TB to prevent premature deaths.

Given that TB is the predominant cause of death among HIV-positive individuals, understanding the pathological mechanisms of HIV-TB coinfection is crucial for identifying potential inflammatory markers (7). Our research focuses on examining levels of inflammation by assessing acute phase proteins to gauge the extent of immunosuppression in HIV-TB coinfected patients and to monitor changes in inflammatory responses before and after Isoniazid Preventive Treatment (IPT). IPT is administered to all HIV-positive patients as a preventative measure against TB.

The acute phase response, a systemic reaction to infection, inflammation, or trauma, prompts the liver to produce elevated levels of certain plasma proteins known as Acute Phase Proteins (8).We hypothesize that the co-occurrence of HIV and latent TB infection (LTBI) leads to a more complex immune response. HIV-induced immunosuppression increases the risk of TB reactivation, while the persistent immune activation from both infections may drive elevated acute phase protein levels. These heightened acute-phase proteins levels could serve as indicators of increased risk for progression from latent to active TB in HIV-infected individuals. By measuring acute-phase proteins levels, we aim to distinguish inflammatory responses between HIV-positive individuals with and without LTBI, potentially providing a biomarker for monitoring disease activity. This study also evaluates the efficacy of IPT in managing latent TB by comparing acute-phase proteins levels before and after treatment. Acute Phase Proteins such as a-2-macroglobulin (A2M), C-Reactive Protein, Serum Amyloid P (SAP), haptoglobin, ferritin, sTFR, apotransferrin, hepcidin, S100A8, and S100A9 are analyzed to potentially establish these markers for early TB prognosis in HIV-positive patients.

## Materials and methods

### Ethics statement

This study received approval from the Ethics Committees of ICMR-NIRT (NIRT IEC No: 2019033, 14^th^ October 2019), the Institutional Ethics Committee of Madras Medical College (IEC No. 31042021, 21^st^ April,2021), and the Institutional Review Board of the Government Hospital of Thoracic Medicine (Approved on 6^th^ February 2021). Informed written consent was secured from all participants.

### Study population

The study was a prospective analysis conducted at the anti-retroviral therapy (ART) centers of the Government Hospital of Thoracic Medicine and Rajiv Gandhi Government General Hospital in Chennai. From April 2021 to November 2022, HIV seropositive individuals were screened for latent tuberculosis (TB) using the QuantiFERON TB Gold in-tube (QGIT) and enrolled prior to the initiation of isoniazid (INH) prophylaxis. All HIV positive were on ART for more than two years. In total, 101 HIV-positive participants were recruited at baseline and categorized based on their latent TB status into two groups: pre-prophylaxis HIV+ with Latent TB (n=30) and pre-prophylaxis HIV+ without Latent TB (n=71) (**Table 1**). Follow-up was conducted on 71 individuals after six months of completing IPT. The loss to follow-up occurred due to participants relocating, making it difficult to contact them, and because some contact details (e.g., phone numbers or addresses) were changed or outdated. Inclusion criteria were HIV seropositive individuals of any CD4 count residing in or around Chennai. Exclusion criteria included symptomatic TB, positive sputum smear or bacteriology for TB, current or past TB treatment, chronic viral hepatitis (HBV/HCV), other active infections, autoimmune diseases, psychiatric or immunological illnesses, or those on corticosteroids, immunosuppressants, or immunomodulatory treatments. Collected blood samples were processed in sodium heparin tubes and transported to the Immunology lab within two hours, with plasma samples subsequently stored at -80°C. We have collected sputum samples from all the enrolled study participants after completion of IPT treatment and none of the patients were found to be active TB after IPT treatment.

**Table 1:** Study demographics.

Serial number	Characteristics of study population	HIV-Positive LTBI-Positive	HIV-Positive LTBI-Negative
1	Sample Size	N=30	N=71
2	Age	43 (19-56)	40 (18-55)
3	Male	20 (30%)	47 (71%)
4	Female	10 (30%)	24 (71%)
5	Height, kg (Median)	162 (150-174)	160 (143-180)
6	Weight, cm (Median)	64.5 (41-95)	60 (37-93)
7	Cough	1 (3.33%)	4 (56.3%)
8	Haemoptysis	0	1 (1.4%)
9	Fever	0	1 (1.4%)
10	Loss of appetite	1 (3.33%)	3 (4.2%)
11	Loss of weight	0	3 (4.2%)
12	Bacillus Calmette-Guerin (BCG) scar	28 (93.3%)	64 (90.1%)
13	Household contact of TB	2 (6.6%)	2 (2.8%)
14	Diabetes Mellitus (DM)	0	3 (4.2%)

*The percentage listed in the table represents the total percentage for each specific parameter mentioned.

### Acute phase protein assays

Plasma levels of alpha-2-macroglobulin (A2M), C-reactive protein (CRP), serum amyloid P (SAP), and haptoglobin were measured using the Milliplex MAP Human CVD Panel Acute Phase magnetic bead panel 3 from (Millipore, Darmstadt, Germany) employing a multiplex platform following the manufacturer’s instructions. The lowest detection limits for acute phase proteins were as follows: A2M, 0.49 ng/mL; CRP, 0.05 ng/mL; haptoglobin, 0.06 ng/mL; and SAP, 0.06 ng/mL. Ferritin, 93.8 pg/mL levels were assessed using the DuoSet ELISA kit (R&D Systems, Minneapolis, USA) with a lowest detection limit of 93 pg/mL, while soluble transferrin receptor (sTFR) levels were determined using a quantitative ELISA kit from Bio Vendor, with a lowest detection limit of 0.05 μg/mL. Apotransferrin and hepcidin levels were measured using quantitative ELISA kits (Cloud Clone Corp., Katy, Texas, USA) with apotransferrin having a lowest detection limit of 0.312 pg/mL and hepcidin of 62.5 pg/mL. S100A8 and S100A9 levels were estimated using DuoSet ELISA kit (R&D Systems, Minneapolis, USA) S100A8 has the lowest detection limit of 31.3 pg/mL and S100A9 of 31.3 pg/mL.

### Latent TB diagnosis

Latent TB infection was diagnosed based on a positive QuantiFERON TB Gold in-tube (QGIT) test with IFN-γ >0.35 IU/ml. All enrolled individuals had no signs or symptoms of active TB, no prior history of TB, and normal chest radiographs. The QGIT was performed according to the manufacturer’s instructions (Qiagen).

### Statistical analyses

Geometric means were used to measure central tendency. Statistically significant differences between the HIV+LTB+ and HIV+LTB- groups were analyzed using the Mann–Whitney U-test, while pre- and post-treatment levels were compared using the Wilcoxon signed-rank test. Data analysis was performed using GraphPad PRISM version 10 (GraphPad Software, Inc., San Diego, CA, USA). Spearman’s correlation was plotted using R Studio, and differences in the expression of each acute phase protein across the groups were visualized and observed using the “Complex Heatmap” package in “RStudio 2023.06.1 + 524.”

## Results

### Elevated acute phase proteins in human immunodeficiency virus infection with latent tuberculosis infection

**Figure 1:**
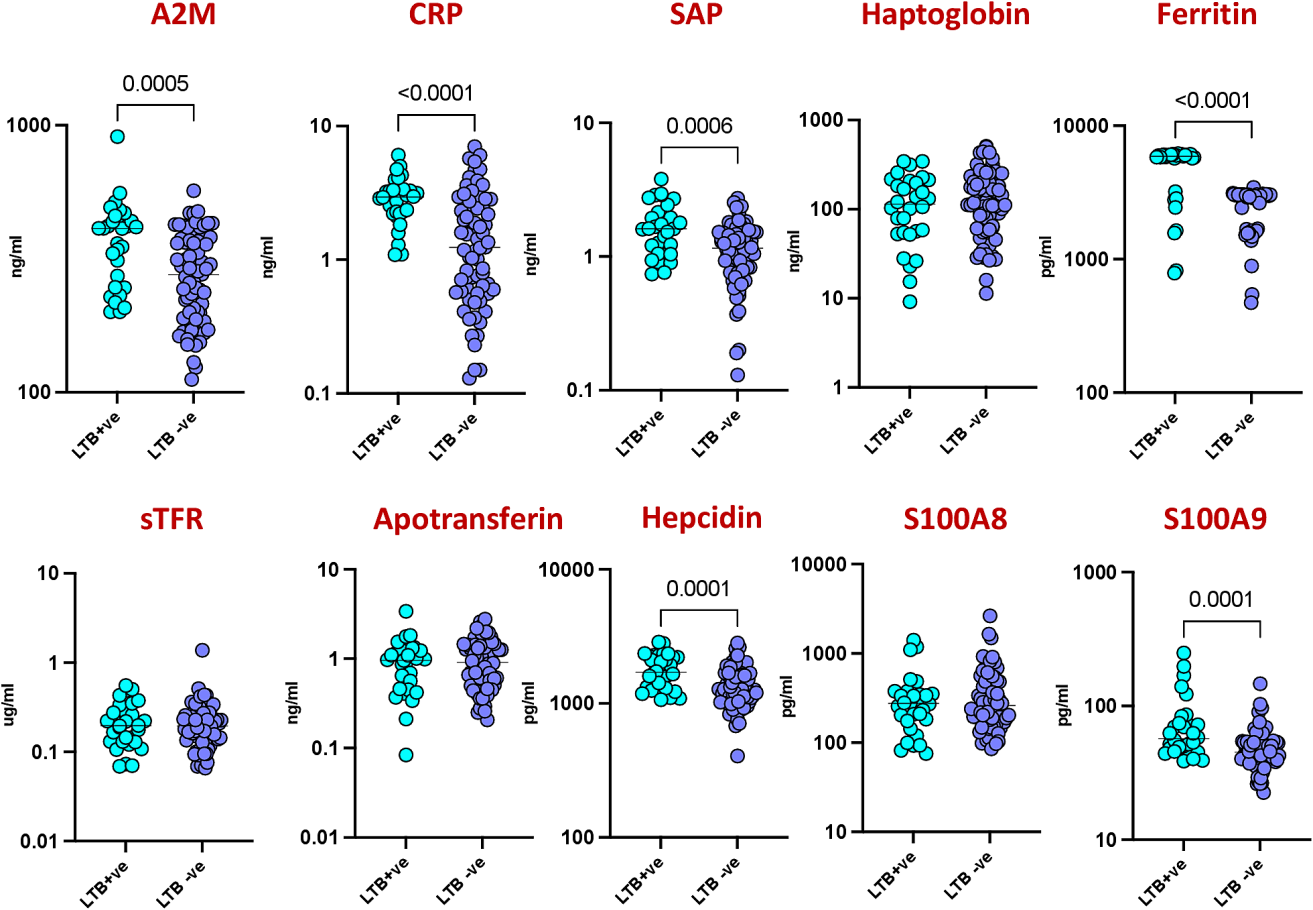
Elevated acute phase proteins in HIV patients with LTBI. Plasma levels of acute phase proteins (APPs) were measured in HIV patients with latent tuberculosis infection (LTBI) (n = 30) and HIV patients without LTBI (n = 71) using multiplex and quantitative ELISA kit. Each circle represents an individual, with p values calculated using the Mann–Whitney test with Holm’s correction for multiple comparisons.

To assess inflammatory responses between latent tuberculosis infection (LTBI) positive and negative groups in HIV-infected patients, we measured the levels of ten different acute phase proteins (APPs). As shown in **Figure 1** Significant elevations were observed in several markers among LTBI positive individuals compared to LTBI negative individuals. Specifically, levels of Alpha-2-Macroglobulin (A2M) (p=0.0005), C-Reactive Protein (CRP) (p<0.0001), Serum Amyloid P (SAP) (p=0.0006), Ferritin (p<0.0001), Hepcidin (p=0.0001), and S100A9 (p=0.0001) were notably higher in the LTBI positive group. These findings indicate that LTBI positive individuals exhibit heightened inflammatory responses compared to LTBI negative individuals.

### Reduced acute phase proteins post-isoniazid preventive therapy in human immunodeficiency virus infection with latent tuberculosis infection

**Figure 2:**
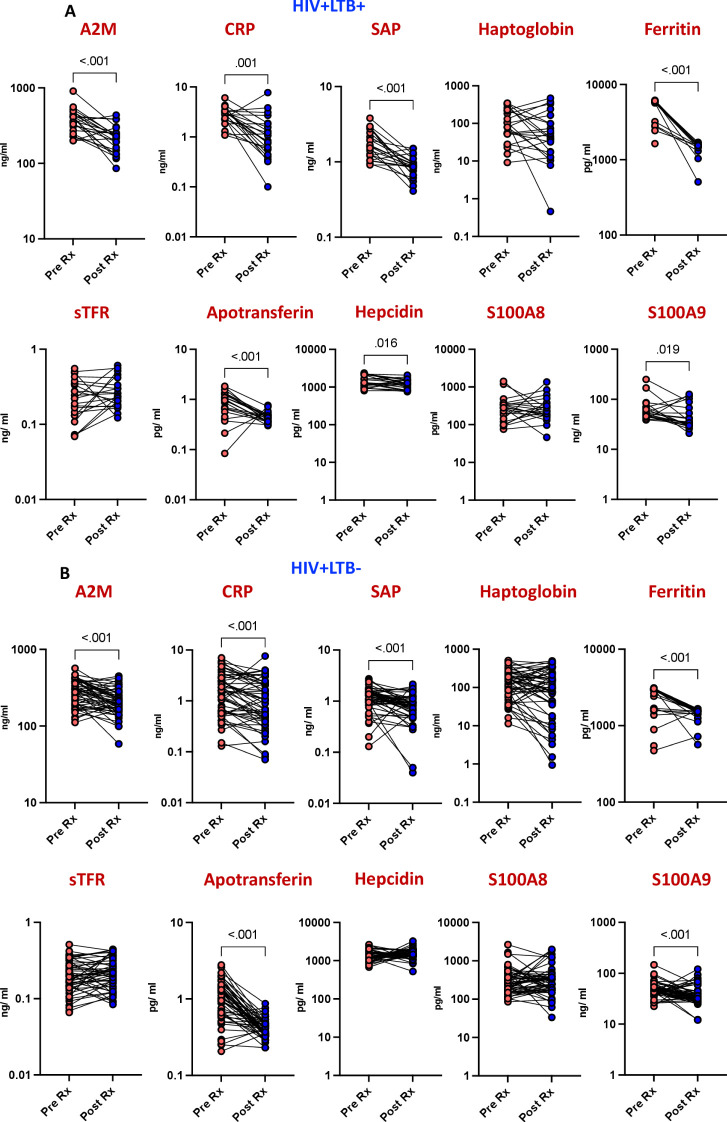
Diminished APPs levels after IPT in HIV patients. The plasma levels of APPs were measured at baseline (pre) and 6 months following isoniazid preventive treatment (IPT) in **(A)** HIV patients with LTBI (n = 22) and **(B)** HIV patients without LTBI (n = 52). Line graphs represent individual trajectories, with p values calculated using the Wilcoxon signed rank test.

To evaluate the effectiveness of isoniazid preventive therapy (IPT) against latent tuberculosis infection (LTBI) in HIV-infected individuals, we compared the levels of acute phase proteins (APPs) before and after IPT treatment. Baseline inflammatory responses and responses six months post-treatment were recorded, compared, and analyzed statistically. As shown in **Figure 2A** the comparison revealed significant decreases in the levels of several markers post-IPT compared to pre-IPT levels. Specifically, A2M (p<0.001), CRP (p=0.001), SAP (p<0.001), ferritin (p<0.001), apotransferrin (p<0.001), hepcidin (p=0.016), and S100A9 (p=0.019) exhibited considerable reductions post-IPT.

### Reduced acute phase proteins post-isoniazid preventive therapy in human immunodeficiency virus infection without latent tuberculosis infection

To assess the impact of isoniazid preventive therapy (IPT) on inflammation in HIV-positive individuals without latent tuberculosis infection (LTBI), we examined the levels of acute phase proteins (APPs) before and after IPT treatment. Comparing pre- and post-IPT levels, a significant decrease was observed in several markers among the LTBI-negative population. Specifically, A2M (p<0.001), CRP (p<0.001), SAP (p<0.001), ferritin (p<0.001), apotransferrin (p<0.001), and S100A9 (p<0.001) exhibited notable reductions post-IPT (**Figure 2B**). These findings underscore the effectiveness of isoniazid in reducing inflammation in HIV-positive individuals without LTBI.

### Distinct plasma acute phase protein profile in human immunodeficiency virus infection with latent tuberculosis infection

**Figure 3:**
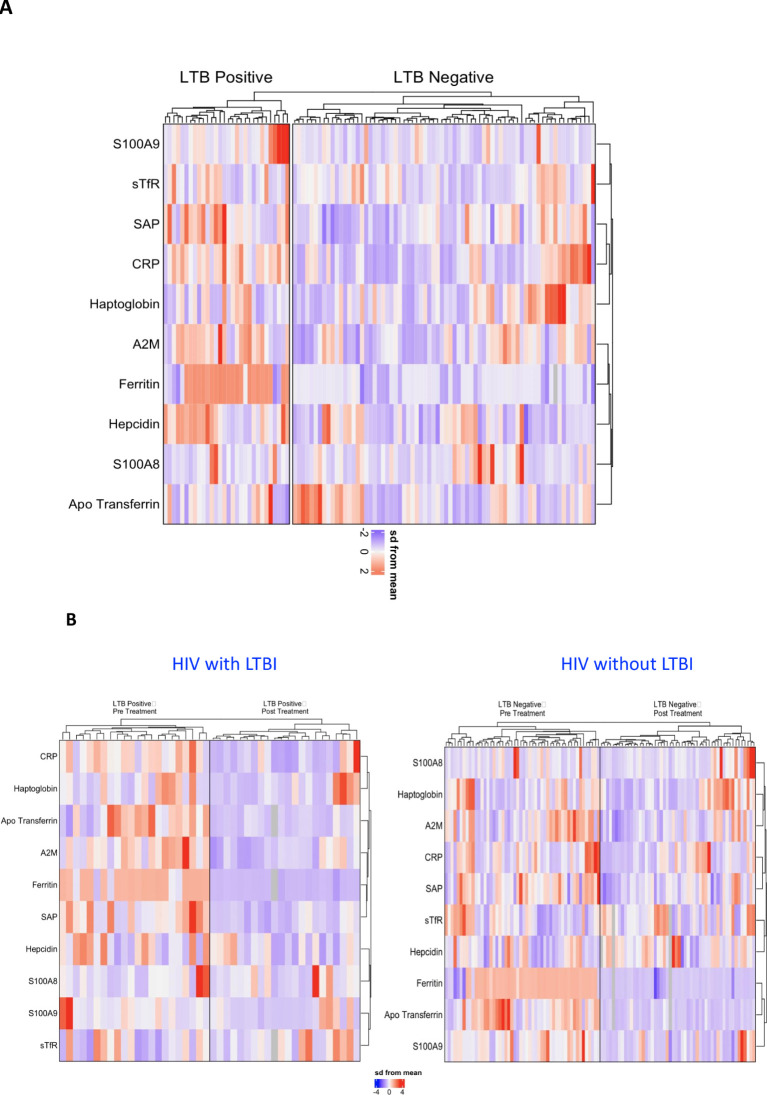
Profiling of APPs as immune markers in HIV patients with and without LTBI. Hierarchical cluster analysis was conducted to examine immune markers that differed statistically between HIV patients with and without LTBI. **(A)** Comparison between HIV patients with and without LTBI, **(B)** HIV patients with LTBI, and HIV patients without LTBI during pre and post IPT. Univariate analysis enabled the separation of groups based on individual levels of each subject. Data were log10 transformed and z-score normalized.

Our investigation aimed to discern a signature pattern of acute phase proteins (APPs) in HIV patients with and without latent tuberculosis infection (LTBI). Analysis of plasma samples revealed statistically significant differences in several APP concentrations between the two study groups. Z-score normalization and hierarchical clustering highlighted distinct clusters, notably indicating a heightened inflammatory response in the LTBI-positive group. Specifically, as shown in **Figure 3A** elevated levels of APPs including A2M, CRP, SAP, Ferritin, Hepcidin, and S100A9 were observed in LTBI-positive individuals compared to LTBI-negative counterparts. This suggests a characteristic systemic profile of APPs that distinguishes LTBI-positive from LTBI-negative patients in our cohort. Furthermore, post-treatment analyses revealed a significant reduction in APP levels in both LTBI-positive and LTBI-negative groups (**Figures 3B**). Interestingly, the magnitude of decrease was comparatively higher in the LTBI-negative group. These findings underscore the efficacy of treatment in mitigating inflammatory responses, particularly pronounced in LTBI-negative individuals.

## Discussion

Systemic inflammation, characterized by elevated acute phase proteins (APPs), serves a hallmark of infection. HIV’s strong predictive value for TB reactivation underscores the necessity of baseline LTBI testing for all HIV-positive patients (8). Our study compared APP levels in LTBI-positive versus LTBI-negative HIV patients, revealing significantly higher levels in the LTBI-positive group, indicating an increased inflammatory response due to HIV-TB coinfection. APPs have shown promise as predictive biomarkers for TB treatment failure.

The latency period in TB is characterized by persistent T cell activation, granuloma formation, and containment of *M. tuberculosis*. Studies have shown increased inflammatory responses in individuals with latent TB compared to those without, with disruptions in granuloma balance potentially reactivating mycobacterial infection and intensifying host inflammatory responses (9–11). The reactivation of mycobacteria reduces host iron content, as evidenced by elevated ferritin and hepcidin levels in LTBI-positive individuals (12).

Our study findings are supported by previous research demonstrating significantly elevated CRP levels in HIV-infected patients with TB, as well as modulation in serum levels of A2M and haptoglobin in TB patients (13). SAP levels have been associated with the innate immunity of the host against bacterial infection, while S100A8/A9 proteins mediate neutrophil accumulation in TB inflammatory granulomas, serving as potential biomarkers for TB severity (14–16).

Preventing TB reactivation and decreasing susceptibility to new *M. tuberculosis* infections are critical factors in reducing mortality from HIV-TB coinfection. IPT treatment has been shown to effectively reduce TB incidence among HIV-positive patients, with our study revealing differences in APP levels pre and post IPT in both HIV-positive populations with and without LTBI. The reduction in inflammatory markers in individuals without latent tuberculosis but treated with isoniazid is indeed an interesting observation. Isoniazid is known to affect the immune system beyond its role in targeting Mycobacterium tuberculosis. It could potentially have immunomodulatory effects that reduce systemic inflammation (17–19). The significant decrease in APP levels post IPT suggests the effectiveness of isoniazid in reducing inflammation and preventing LTBI reactivation.

In our study, we also observed that two acute-phase proteins, apotransferrin and hepcidin, are differentially regulated following the completion of IPT treatment in both LTBI-positive and LTBI-negative individuals. Previous studies have reported that both HIV and TB infections can disrupt iron metabolism, potentially leading to a state of “functional iron deficiency,” where iron is present in the bloodstream but is not properly utilized due to alterations in iron-regulating proteins such as ferritin and transferrin. This dysfunction can impact immune function, as iron is essential for the activity of immune cells, particularly macrophages, which play a key role in combating *M. tuberculosis* (20).

Hepcidin, in particular, is crucial in regulating iron availability and modulating immune responses. The interplay between iron regulation and immune responses in the context of HIV and TB has significant implications for understanding disease progression and developing effective interventions (20, 21). However, our study has certain limitations, including a small sample size and the absence of a healthy control group.

In conclusion, our study highlights the positive correlation between APP levels and inflammation levels in the host, with different APP levels aiding in the differentiation of LTBI-positive and negative individuals and the assessment of IPT efficacy. A key strength of this study is its focus on identifying early biomarkers that can monitor effectiveness of prophylactic anti-TB treatment. These findings contribute to our understanding of HIV-TB coinfection pathogenesis and the role of IPT, offering valuable insights into potential biomarkers for improved diagnosis and treatment monitoring.

## Data Availability

The original contributions presented in the study are included in the article/**Supplementary Material**. Further inquiries can be directed to the corresponding authors.
