# Supplementary material for: Acute-phase proteins as biomarkers of inflammation in HIV patients with latent tuberculosis: a prospective study
Source: Front Immunol. 2025 Apr 30;16:1551775. doi: 10.3389/fimmu.2025.1551775 (PMC12075211; doi:10.3389/fimmu.2025.1551775)
Supplement: Supplementary file 1 [file DataSheet1.pdf]

Sup Table 1: HIV-Positive LTBI-Positive

Parameters	A2M		CRP		SAP		Haptoglobin		Ferritin	
	Pre Rx	Post Rx	Pre Rx	Post Rx	Pre Rx	Post Rx	Pre Rx	Post Rx	Pre Rx	Post Rx
pValue	<0.001		0.001		<0.001		0.483		<0.001	
Mean	388	225.5	2.87	1.501	1.829	0.8977	127.5	101.7	5201	1474
Lower 95% CI of mean	319.1	184.5	2.363	0.755	1.472	0.7663	78.9	43.29	4544	1356
Upper 95% CI of mean	457	266.5	3.376	2.247	2.187	1.029	176.2	160.1	5858	1593
25% Percentile	265.8	145.3	2.218	0.53	1.17	0.6675	46.06	16.08	5078	1486
Median	385.8	223.2	2.96	0.955	1.61	0.87	90.52	44.01	5967	1547
75% Percentile	447.9	265.6	3.303	1.84	2.51	1.01	216.6	115.4	6055	1583
Parameters	**sTFR**		**Apotransferin**		**Hepcidin**		**S100A8**		**S100A9**	
	Pre Rx	Post Rx	Pre Rx	Post Rx	Pre Rx	Post Rx	Pre Rx	Post Rx	Pre Rx	Post Rx
pValue	0.149		<.001		0.016		0.824		0.019	
Mean	0.2514	0.2892	0.9311	0.4476	1529	1253	343.7	331.3	68.65	48.61
Lower 95% CI of mean	0.1863	0.2247	0.7163	0.3916	1288	1093	197.6	201.5	47	34.79
Upper 95% CI of mean	0.3164	0.3538	1.146	0.5036	1770	1413	489.9	461.1	90.29	62.43
25% Percentile	0.131	0.1805	0.5348	0.355	1063	926	137.6	170.9	45.5	29.75
Median	0.221	0.237	0.974	0.42	1319	1233	280.4	233.8	51.78	33.67
75% Percentile	0.3565	0.375	1.315	0.5	2139	1531	353.4	374.8	69.51	56.46

Sup Table 2: HIV-Positive LTBI-Negative

Parameters	A2M		CRP		SAP		Haptoglobin		Ferritin	
	Pre Rx	Post Rx	Pre Rx	Post Rx	Pre Rx	Post Rx	Pre Rx	Post Rx	Pre Rx	Post Rx
pValue	<0.001		<0.001		<0.001		0.439		<0.001	
Mean	288.3	218.5	1.98	1.427	1.233	0.8838	160.8	148.7	2677	1467
Lower 95% CI of mean	257.8	195.2	1.511	1.029	1.08	0.7631	125.7	110.5	2486	1385
Upper 95% CI of mean	318.7	241.7	2.449	1.824	1.386	1.005	195.8	187	2868	1550
25% Percentile	189.4	165.7	0.605	0.405	0.935	0.595	58.54	39.19	2911	1494
Median	280.3	204	1.57	0.95	1.265	0.885	131.9	104.9	2978	1539
75% Percentile	379.4	253.4	2.815	2.208	1.535	1.105	219.9	222.7	3021	1577
**Parameters**	**sTFR**		**Apotransferin**		**Hepcidin**		**S100A8**		**S100A9**	
	Pre Rx	Post Rx	Pre Rx	Post Rx	Pre Rx	Post Rx	Pre Rx	Post Rx	Pre Rx	Post Rx
pValue	0.266		<0.001		0.272		0.989		<0.001	
Mean	0.2152	0.2346	1.094	0.4234	1429	1585	397.5	426.5	50.47	40.66
Lower 95% CI of mean	0.1873	0.2047	0.9256	0.3871	1303	1448	274.9	295.5	45.01	35.15
Upper 95% CI of mean	0.2431	0.2645	1.262	0.4597	1554	1722	520.2	557.5	55.92	46.16
25% Percentile	0.144	0.1545	0.6113	0.33	1122	1316	154.1	172.3	41.75	29.79
Median	0.198	0.2165	1.02	0.41	1309	1504	261.5	250.4	45.12	33.17
75% Percentile	0.2743	0.296	1.427	0.4725	1683	1870	484.9	406.2	53.86	44.13
